# Characterization of the *Enterobacter* Phage vB_EclM_CIP9

**DOI:** 10.1128/MRA.01600-19

**Published:** 2020-03-26

**Authors:** Klara Wang, Marielou G. Tamayo, Tiffany V. Penner, Bradley W. M. Cook, Deborah A. Court, Steven S. Theriault

**Affiliations:** a Cytophage Technologies, Inc., Winnipeg, Manitoba, Canada; b Department of Microbiology, University of Manitoba, Winnipeg, Manitoba, Canada; DOE Joint Genome Institute

## Abstract

Enterobacter cloacae is an opportunistic pathogen that causes hospital-acquired infections in immunocompromised patients. Here, we describe vB_EclM_CIP9, a novel *Enterobacter* phage that infects a multidrug-resistant isolate of E. cloacae. Phage vB_EclM_CIP9 is a myovirus that has a 174,924-bp genome, with 296 predicted open reading frames.

## ANNOUNCEMENT

Most clinically relevant Enterobacter cloacae isolates are resistant to select β-lactam antibiotics, including ampicillin and amoxicillin. Antibiotic resistance limits treatment options to control E. cloacae infections in immunocompromised patients and can lead to severe health problems such as bacteremia, endocarditis, and/or death (1, 2). Lytic bacteriophages hold a potential solution to the problem posed by antibiotic resistance (3). The objective of this study was to characterize the phage vB_EclM_CIP9, with specificity against a clinical isolate of E. cloacae.

**FIG 1:**
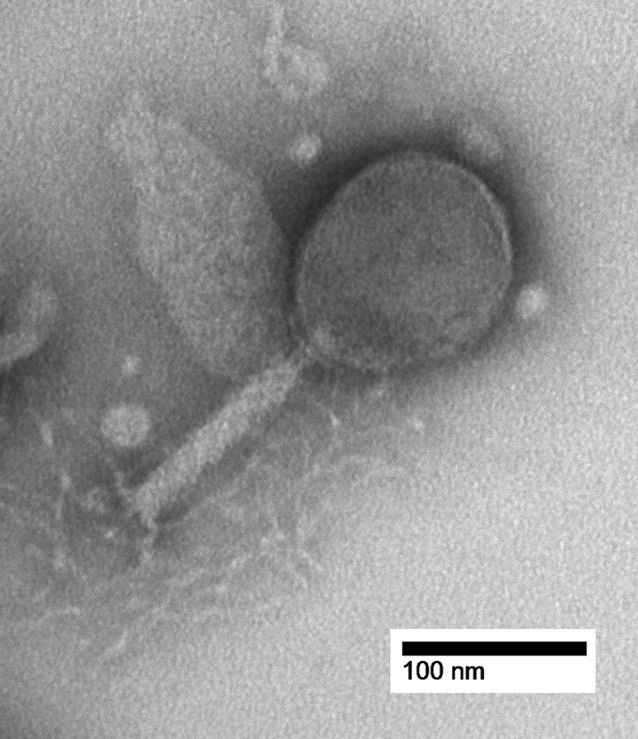
Transmission electron micrograph of vB_EclM_CIP9. Purified vB_EclM_CIP9 phage particles were stained with 2% uranyl acetate. Images were obtained with a Hitachi H7600 transmission electron microscope and AMT XR51 charge-coupled device camera at 80 kV, at a magnification of ×200,000.

*Enterobacter* phage vB_EclM_CIP9 was isolated in 2017, from a municipal wastewater sample, against a clinical E. cloacae isolate. Briefly, E. cloacae was grown on tryptic soy broth or agar (Becton, Dickinson and Company) at 37°C with aeration. The wastewater sample was centrifuged and filtered. Ten milliliters of the clarified wastewater was mixed with 200 μl of E. cloacae grown to an optical density (at 600 nm) of 0.7, and the mixture was incubated overnight to enrich for E. cloacae-specific phages (4). Subsequent plaque purification and phage propagation were conducted by the soft-agar overlay method (5, 6). High-titered phage (>10^8^ PFU/ml) was purified with 20% sucrose (7) and visualized by electron microscopy (Bioimaging Facility, University of British Columbia, Vancouver, British Columbia, Canada). Phage vB_EclM_CIP9 has a *Myoviridae* morphology, with an average head size of 132 ± 2 nm and tail size of 119 ± 1 nm, as measured from 3 independent images (Fig. 1).

The genomic contents from plaque-purified phage particles were extracted (PureLink viral RNA/DNA minikit; Thermo Fisher Scientific, Ontario, Canada), independently treated with DNase I (1 μg/ml) and RNase (1 μg/ml) (New England BioLabs, Ontario, Canada), and analyzed by agarose gel electrophoresis to determine the identity of the nucleic acids in the sample. The genomic DNA was prepared for sequencing (TruSeq Nano DNA sample preparation kit; Illumina, San Diego, CA) with the MiSeq 2000 platform (2 × 300-bp reads using MiSeq reagent kit v3 chemistry; Illumina) from an average fragment length of 500 bp (National Research Council Canada, Saskatoon, Saskatchewan, Canada). After quality control with FastQC v0.11.8 (8), the 1,585,393 paired-end reads were *de novo* assembled (Geneious Prime v2019.2.3) to yield a contig of 174,924 bp (53-fold coverage), with a GC content of 39.9%. The genome was annotated with Rapid Annotations using Subsystems Technology (RAST) v2.0 (9) and Phage Search Tool Enhanced Release (PHASTER) (10). All predicted open reading frames (ORFs) were subjected to a Basic Local Alignment Search Tool (BLAST) search (11). All software programs used in this study were run with default parameters.

The vB_EclM_CIP9 genome is circularly permuted and terminally redundant (PhageTerm) (12). There are 296 predicted ORFs, with 253 on one strand and the remaining 43 on the opposite strand. BLAST (11) analysis revealed 114 putative ORFs coding for common phage gene products with assigned functions. No ORFs were found to be associated with virulence factors, antibiotic resistance genes, toxins, or integration elements (PHASTER) (10). When the genome of vB_EclM_CIP9 was compared with complete phage genomes with BLAST (11), the results indicated that the genome of vB_EclM_CIP9 exhibited a nucleotide alignment of only 74% and a nucleotide identity of 81.15%, compared with the genome of the *Edwardsiella* phage PEi20 (GenBank accession number NC_028683). Similarly, the genome of vB_EclM_CIP9 exhibited a nucleotide alignment of only 73% and a nucleotide identity of 81.13%, compared with the genome of the *Edwardsiella* phage PEi26 (GenBank accession number AP014715.1).

### Data availability.

The genome sequence and associated data for phage vB_EclM_CIP9 were deposited under GenBank accession number MN882610, BioProject accession number PRJNA608533, SRA accession number SRR11178671, and BioSample accession number SAMN14177620.
